# Sex and hibernaculum temperature predict survivorship in white-nose syndrome affected little brown myotis (*Myotis lucifugus*)

**DOI:** 10.1098/rsos.140470

**Published:** 2015-02-04

**Authors:** Laura E. Grieneisen, Sarah A. Brownlee-Bouboulis, Joseph S. Johnson, DeeAnn M. Reeder

**Affiliations:** Department of Biology, Bucknell University, Lewisburg, PA 17837, USA

**Keywords:** Chiroptera, disease ecology, *Pseudogymnoascus destructans*

## Abstract

White-nose syndrome (WNS), an emerging infectious disease caused by the novel fungus *Pseudogymnoascus destructans*, has devastated North American bat populations since its discovery in 2006. The little brown myotis, *Myotis lucifugus*, has been especially affected. The goal of this 2-year captive study was to determine the impact of hibernacula temperature and sex on WNS survivorship in little brown myotis that displayed visible fungal infection when collected from affected hibernacula. In study 1, we found that WNS-affected male bats had increased survival over females and that bats housed at a colder temperature survived longer than those housed at warmer temperatures. In study 2, we found that WNS-affected bats housed at a colder temperature fared worse than unaffected bats. Our results demonstrate that WNS mortality varies among individuals, and that colder hibernacula are more favourable for survival. They also suggest that female bats may be more negatively affected by WNS than male bats, which has important implications for the long-term survival of the little brown myotis in eastern North America.

## 2. Introduction

White-nose syndrome (WNS) is an emerging infectious disease estimated to have killed over 5.7 million North American bats [1] in the 4 years following its discovery. It is caused by the psychrophilic fungus, *Pseudogymnoascus destructans* (*Pd*) [2] that grows in the skin of affected bats during periods of prolonged torpor, or hibernation [3]. At least seven species of bats are affected, but little brown myotis (*Myotis lucifugus*) are especially susceptible, with an average 91% decline in northeastern North America [4]. WNS affects hibernation behaviour and survival in bats [2,5]; the role of microclimate factors, such as hibernaculum temperature, on this relationship is mostly unknown, although populations of little brown myotis in warmer WNS-affected hibernacula suffer greater declines than those in colder hibernacula [6]. In healthy bats, hibernaculum temperature plays a significant role in energy balance and survivorship [7]. When a bat enters torpor its metabolism is depressed and body temperature drops to within 1°C of ambient temperature [8]. Hibernators use up to 90% of their stored energy for arousal bouts [9], in which they briefly warm up to euthermic body temperature. Arousing from a warmer temperature uses less energy than arousing from a colder temperature, thus bats roosting at warmer temperatures can arouse more frequently [7]. Sex also plays a role in hibernation energetics; female bats face selective pressure to retain enough fat at the end of hibernation to ovulate upon spring emergence [10,11].

The goal of this study was to determine the impact of hibernaculum temperature and other covariates such as sex and body mass index (BMI) on WNS survivorship in little brown myotis in controlled laboratory studies. Because *Pd* grows optimally between 12.5°C and 15.8°C, and much more slowly at lower temperatures [12] we hypothesized that WNS-affected bats housed at colder temperatures would exhibit increased survival over those housed at warmer temperatures. We further hypothesized that females infected with *Pd* would have increased survival rates over males, as healthy female little brown myotis start and end hibernation with greater fat reserves [11], and may be better equipped to deal with an energetically costly disease during hibernation.

## 3. Material and methods

### 3.1 Study 1

Little brown myotis were collected from WNS-unaffected (*n*=58) and WNS-affected (*n*=49) hibernacula in Pennsylvania on 13 and 15 January 2010, respectively, and transported to Bucknell University's bat vivarium. Every bat from the WNS site had visible fungal growth at collection and was thus presumed to be WNS-affected. Upon arrival at the laboratory, data on weight, sex and forearm length were recorded for each bat and BMI (mass in g/length of forearm in mm) was calculated. Bats were housed following [13] and induced to hibernate by placing them in a darkened environmental chamber. Bats were evenly distributed between six non-adjacent wire mesh cages (46×46×61 cm); one for unaffected bats, one for WNS-affected bats in each of three environmental chambers (set to 4°C, 7°C or 10°C; table 1). Lorch *et al*. [14], under nearly identical housing conditions, demonstrated that *Pd* transmission does not occur between cages, and, as visible fungal growth was not observed on the unaffected bats at any time in our study it is unlikely transmission occurred between the groups. Chambers were checked weekly for mortality and dead bats were removed. This provided a relative death date, so survival could be calculated via Cox regression on the following variables: WNS status of a site, chamber temperature, sex and initial BMI. Additionally, a subset of animals from each group were removed once for experimental testing of thermal preferences for a separate study. While these trials were conducted with affected and unaffected bats equally, this disturbance, combined with weekly removal of dead animals, very likely affected overall survivorship for all bats (see Results).

**Table 1.** Factors included in the study 1 final Cox survival regression model. (A hazard ratio of 0.353 indicates a 35.3% lower risk of death. Asterisks indicate significance of *p*-values.)

effect	sample size, *n*	*χ*^2^	*p*-value	parameter	group with higher risk of death	hazard ratio (95% CI)
site*	WNS site: 58	11.70	<0.001*	—	WNS-affected	—
	unaffected site: 49					
sex	female: 55	0.10	0.75	—	—	—
	male: 52					
sex×site*	WNS female: 24 unaffected female: 31 WNS male: 24 unaffected male: 28	4.26	0.04*	WNS male versus WNS female	WNS female	0.468 (0.253–0.866)
				unaffected male versus unaffected female	n.s.	1.090 (0.647–1.837)
				unaffected female versus WNS female	WNS female	0.152 (0.080–0.287)
				unaffected male versus WNS male	WNS male	0.353 (0.195–0.641)
housing temperature*	4°C: 38 7°C: 34 10°C: 35	11.17	0.004*	4°C versus 7°C	7°C	1.986 (1.204–3.274)
				4°C versus 10°C	10°C	2.276 (1.360–3.810)
				7°C versus 10°C	n.s.	0.872 (0.537–1.416)
BMI initial	107	3.07	0.08	—	—	—

### 3.2 Study 2

**Figure 1.**
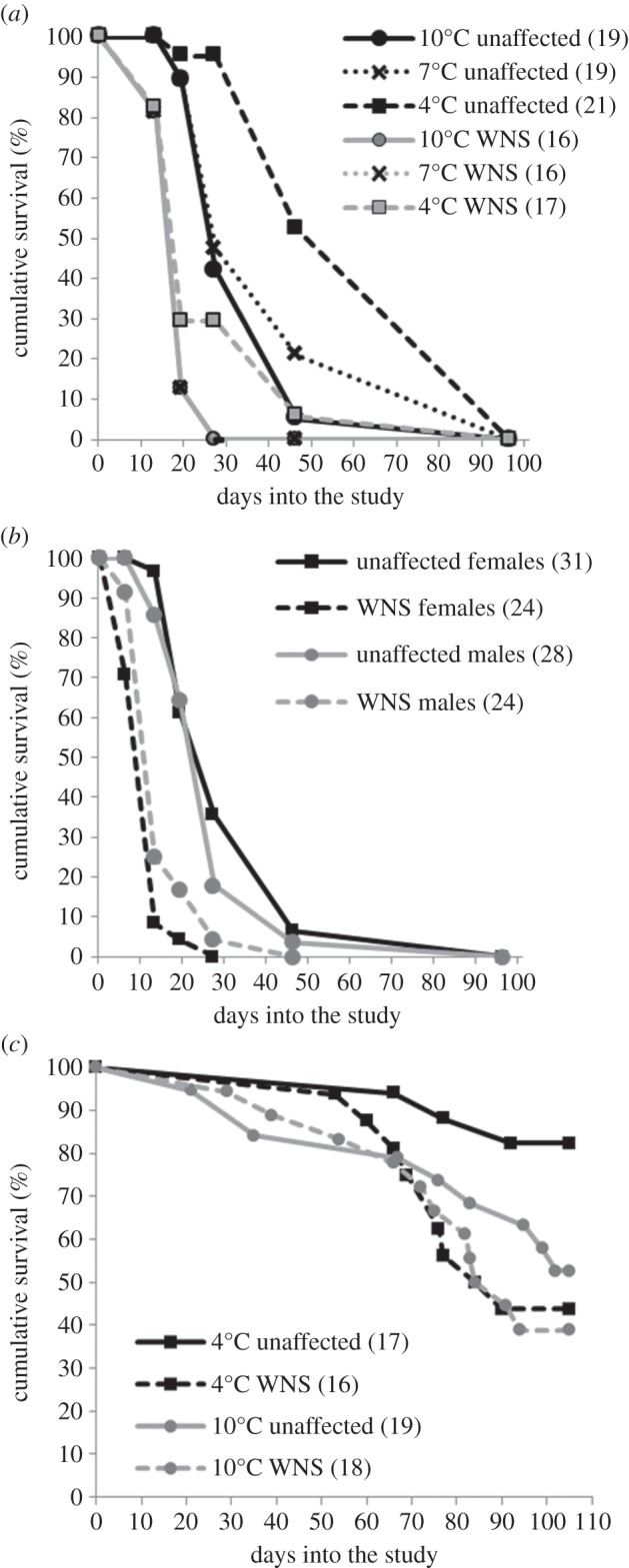
Cumulative survival by WNS status, temperature and sex. (*a*) In study 1, bats from a WNS site and bats housed at 7°C or 10°C had the highest risk of death. (*b*) In study 1, females from a WNS site had a higher risk of death than WNS site males. (*c*) In study 2, bats from a WNS site housed at 4°C had a higher risk of death than bats from an unaffected site. Sample size, *n*, in parentheses.

To follow up on the results of study 1, little brown myotis were collected from a presumed unaffected hibernaculum in Kentucky (*n*=40) and from a WNS-affected hibernaculum (*n*=40) in Pennsylvania on 15 and 21 December 2010, respectively. Collection of animals and research in Pennsylvania was conducted under a Pennsylvania Game Commission permit to DMR (no. 183–2010). Collection of animals from Kentucky was conducted by a state wildlife official (Brooke Hines) on non-endangered bats; thus a numbered permit was not required or issued. Although the presence of *Pd* was detected on a bat from the unaffected site in April 2011, no mortality was detected at the site through spring 2012, strongly suggesting the bats used in this study were unaffected [13]. In order to avoid disturbing more bats than necessary, bats were collected regardless of sex, resulting in a final dataset that was too heavily male-biased (77%) to include sex in the analysis. Bats were processed and housed as described in study 1, and evenly distributed between 4°C and 10°C chambers (table 2). Each bat was fitted with a temperature-sensitive data logger to record its skin temperature (*T*_skin_; [5]) at 30 min intervals. Because of high mortality during study 1, bats remained undisturbed until 23 March 2011. Dead bats were removed and live bats continued to hibernate until 4 April 2011. As weekly mortality therefore could not be calculated, approximate date of death, defined as date of last arousal bout based on *T*_skin_ data, was calculated for those bats whose dataloggers did not malfunction (*n*=70; figure 1*c*). Survival was calculated via Cox regression on WNS status of a site and chamber temperature. Additionally, a Pearson's *χ*^2^ was used to analyse how site and temperature predicted mortality, defined as whether or not a bat survived, for the full dataset (*n*=80).

**Table 2.** Factors included in the study 2 final Cox survival regression model. (Asterisks indicate significance of *p*-values.)

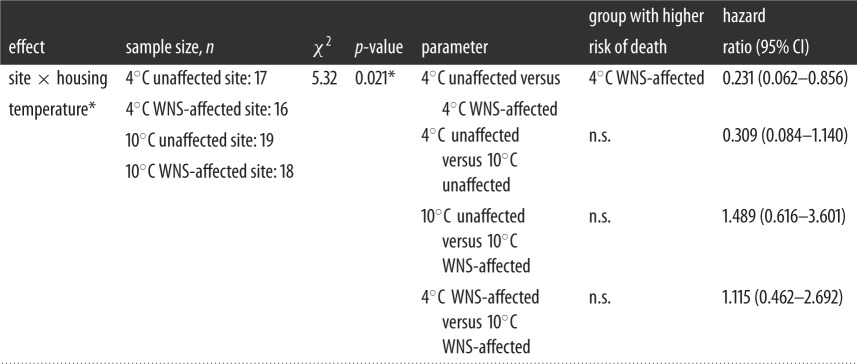

## 4. Results

### 4.1 Study 1

The factors included in the final survival model were WNS status of a site, sex, a WNS status of a *site*×*sex* interaction term, housing temperature and initial BMI. Initial BMI did not have a significant impact on survival, nor did it differ between bats from WNS-affected and unaffected sites (table 1). Temperature had a significant effect on survival; bats housed at 4°C had a significantly lower risk of death than those housed at 7°C or 10°C (table 1 and figure 1*a*). WNS status of a site and the interaction between site and sex also were significant; bats from the WNS site had significantly lower survival than those from the unaffected site, and WNS site female bats had a higher risk of death than WNS site male bats (table 1 and figure 1*b*), even though females had higher initial BMI than males (two-sample *t*-test; *t*_105_=−2.32, *p*=0.023).

### 4.2 Study 2

A hibernation chamber *temperature*×*WNS* status of a site interaction term was the only factor in the Cox survival model. WNS-affected bats housed at 4°C had a significantly higher risk of death than unaffected bats (table 2 and figure 1*c*). Likewise, bats from the WNS site had higher mortality than those from the unaffected site (40% mortality in unaffected bats and 65% mortality in WNS-affected bats; $\chi_{1}^{2}=5.01$, *p*=0.025; table 3), which was primarily driven by differences in mortality in bats housed at 4°C (30% mortality in unaffected bats and 65% mortality in WNS-affected bats; $\chi_{1}^{2}=4.91$, *p*=0.027; table 3).

**Table 3.** Study 2 mortality by treatment group.

group	sample size, *n*	mortality (%, *n*)
4°C unaffected	20	30% (7)
4°C WNS	20	65% (13)
10°C unaffected	20	50% (10)
10°C WNS	20	65% (13)

## 5. Discussion

This is, to our knowledge, the first captive study to test how hibernaculum temperature and sex affect hibernation survival in naturally WNS-affected versus unaffected bats. WNS status of a site, housing temperature and sex all significantly influenced survival during hibernation. The role of WNS status in survival was consistent in both studies; WNS-affected bats fared worse than unaffected bats. However, the impact of housing temperature was more nuanced. Study 1 found that bats housed at a colder temperature fared better than those housed at warmer temperatures, whereas study 2 found unaffected bats fared significantly better than WNS-affected bats at a colder temperature but not at a warmer temperature (at which *Pd* exhibits faster growth [12]). These results suggest that WNS may alter the importance of hibernaculum temperature in surviving hibernation. This could be due to changes in energetic expenditure by WNS-affected bats, perhaps by increasing the amount of time spent grooming and in other expensive behaviours [13], as well as a disruption in physiology [15]. The differences between study 1 and study 2 are probably owing to: (i) the inherent variation in the physiology of captured free-ranging bats, and (ii) the fact while we only collected bats from the affected site that had visible fungal growth on their muzzles and wings, we could not in any way control for infection load or disease stage. Owing to this variation, our results should be interpreted with caution. Truly teasing apart the interaction between *Pd* growth dynamics and temperature on WNS survivorship requires experimental studies in which initial fungal load is controlled. Indeed, we [16] performed these exact experiments in captivity by inoculating naive bats with known fungal loads and housing them at two hibernation temperatures. As in this study in which bats were naturally WNS-affected, a significant effect of hibernation temperature on survivorship in affected bats was found, even after controlling for the overall benefits of colder hibernacula on survivorship. That we have now demonstrated this effect in at least some naturally affected bats and in experimentally inoculated bats highlights the importance of hibernacula temperature on survival in the face of WNS. The finding that colder temperatures are protective for bats suggests that hibernacula temperature modification (especially of mines) may be a viable mitigation strategy for curbing WNS and that protection of colder hibernacula should be prioritized.

This is, to our knowledge, the first study to show that there are sex differences in WNS survival in naturally affected bats, with female bats at greater risk of dying than males. However, these results are not congruent with what would be expected if sex differences in survival were based on differences in energy distribution between males and females. Our study and past studies have found that females start hibernation with higher BMI than males [11,17], which should bias females towards increased survivability. Indeed, our subsequent study under controlled experimental conditions with known *Pd* inoculation doses found significantly greater survivorship in females [16]. Clearly, additional studies of sex biases in the response to *Pd* infection are needed, including those that go beyond mortality estimates and focus on consequences such as metabolic and fitness costs in both males and females that survive WNS.

## Data accessibility

Data are openly published in the Dryad Repository at: [18].
